# Combining next-generation indoor residual spraying and drug-based malaria control strategies: observational evidence of a combined effect in Mali

**DOI:** 10.1186/s12936-020-03361-y

**Published:** 2020-08-15

**Authors:** Joseph Wagman, Idrissa Cissé, Diakalkia Kone, Seydou Fomba, Erin Eckert, Jules Mihigo, Elie Bankineza, Mamadou Bah, Diadier Diallo, Christelle Gogue, Kenzie Tynuv, Andrew Saibu, Jason H. Richardson, Christen Fornadel, Laurence Slutsker, Molly Robertson

**Affiliations:** 1 PATH, Washington, DC USA; 2 Programme National de Lutte contre le Paludisme, Bamako, Mali; 3 RTI International, Washington, DC USA; 4 PMI, USAID, Bamako, Mali; 5 Abt Associates, Bamako, Mali; 6 MEASURE Evaluation, Bamako, Mali; 7 IVCC, Accra, Ghana; 8 IVCC, Washington, DC USA; 9 PATH, Seattle, WA USA

**Keywords:** Indoor residual spraying, Observational analysis, Seasonal malaria chemoprevention, Combined malaria control strategies

## Abstract

**Background:**

Ségou Region in central Mali is an area of high malaria burden with seasonal transmission. The region reports high access to and use of long-lasting insecticidal nets (LLINs), though the principal vector, *Anopheles gambiae*, is resistant to pyrethroids. From 2011 until 2016, several high-burden districts of Ségou also received indoor residual spraying (IRS), though in 2014 concerns about pyrethroid resistance prompted a shift in IRS products to a micro-encapsulated formulation of the organophosphate insecticide pirimiphos-methyl. Also in 2014, the region expanded a pilot programme to provide seasonal malaria chemoprevention (SMC) to children aged 3–59 months in two districts. The timing of these decisions presented an opportunity to estimate the impact of both interventions, deployed individually and in combination, using quality-assured passive surveillance data.

**Methods:**

A non-randomized, quasi-experimental time series approach was used to analyse monthly trends in malaria case incidence at the district level. Districts were stratified by intervention status: an SMC district, an IRS district, an IRS + SMC district, and control districts that received neither IRS nor SMC in 2014. The numbers of positive rapid diagnostic test (RDT +) results reported at community health facilities were aggregated and epidemiological curves showing the incidence of RDT-confirmed malaria cases per 10,000 person-months were plotted for the total all-ages and for the under 5 year old (u5) population. The cumulative incidence of RDT + malaria cases observed from September 2014 to February 2015 was calculated in each intervention district and compared to the cumulative incidence reported from the same period in the control districts.

**Results:**

Cumulative peak-transmission all-ages incidence was lower in each of the intervention districts compared to the control districts: 16% lower in the SMC district; 28% lower in the IRS district; and 39% lower in the IRS + SMC district. The same trends were observed in the u5 population: incidence was 15% lower with SMC, 48% lower with IRS, and 53% lower with IRS + SMC. The SMC-only intervention had a more moderate effect on incidence reduction initially, which increased over time. The IRS-only intervention had a rapid, comparatively large impact initially that waned over time. The impact of the combined interventions was both rapid and longer lasting.

**Conclusion:**

Evaluating the impact of IRS with an organophosphate and SMC on reducing incidence rates of passive RDT-confirmed malaria cases in Ségou Region in 2014 suggests that combining the interventions had a greater effect than either intervention used individually in this high-burden region of central Mali with pyrethroid-resistant vectors and high rates of household access to LLINs.

## Background

For many national malaria control and elimination programmes, decisions about if, where, and when to deploy specific interventions are becoming increasingly complex. The core components of most programmes align with the World Health Organization (WHO) Global Technical Strategy for Malaria 2016–2030 [1] and include strategies to create and maintain universal access to vector control (most commonly long-lasting insecticidal nets [LLINs]), chemoprevention (most commonly intermittent preventive treatment in pregnancy), and accurate diagnosis and appropriate treatment. Since 2000, widespread implementation of these core strategies has contributed substantially to global reductions in malaria morbidity and mortality [2]. However, effective supplementary interventions are becoming more widely available and—in the face of stalled progress and growing concerns about insecticide resistance—more important [1, 3, 4]. This situation naturally leads to questions about how to best layer new tools on top of existing ones to maximize programme impact across various transmission settings [5].

Two additional strategies, both with WHO policy recommendations, are (1) indoor residual spraying (IRS) [6], including the use of third-generation IRS products (3GIRS; products defined as insecticide formulations that are effective at controlling pyrethroid-resistant mosquitoes for at least 6 months) and (2) seasonal malaria chemoprevention (SMC) with sulfadoxine-pyrimethamine plus amodiaquine (SP + AQ) in children under 5 years of age [7]. IRS involves coating the walls and other interior surfaces of a house with a residual insecticide to kill mosquitoes that come in contact with these surfaces [8]. Though IRS can impact vector behaviors and life traits in complex ways, its most important function is usually thought to be its killing action, which decreases the likelihood that mosquitoes survive long enough to become infectious, thereby preventing transmission of malaria. SMC, on the other hand, targets malaria parasites in the human host. It involves the intermittent administration of up to four full treatment courses of an anti-malarial medicine during the malaria season in areas of highly seasonal transmission. When taken as advised, these monthly treatments maintain therapeutic drug concentrations in the blood during the peak transmission period, thereby preventing malaria illness in the target population [9].

Mounting evidence that these two strategies can be effective is encouraging, and each is a welcome addition to the malaria control toolbox [1]. Malaria programmes have used IRS to effectively control, and in some places eliminate, malaria since the 1950s [10]. More recently, the introduction of Actellic® 300CS (Syngenta AG, Basel, Switzerland), a 3GIRS organophosphate product, has been associated with significant reductions in malaria burden in several regions with insecticide-resistant vectors [11–14]. Similarly, the ability of SMC to safely and effectively prevent clinical cases of malaria in young children under trial conditions in areas with seasonal transmission has been well established, even in areas with high net use [15, 16]. Based on this evidence, in 2012 the WHO recommended SMC for children aged 3–59 months in countries of the Sahel sub-region [7]. Following this policy recommendation, eligible countries moved quickly to roll out SMC programmes and by 2017, nearly 16 million children were protected across 12 countries [17]. Though not without challenges, some of which include communicating the need to provide medications to children devoid of symptoms [18] and maintaining high coverage of four treatment rounds [19], implementing SMC as part of a routine malaria control strategy can have a substantial impact on reducing both parasite prevalence and anemia in the targeted age group [20–22]. Additional trials in Ghana and Senegal indicate that the expansion of SMC could be effective in areas with extended transmission seasons [23] and in children over the age of 5 years [24].

Potential exists for an enhanced effect when combining a vector control intervention like 3GIRS and a parasite control intervention like SMC in the same communities at the same time: simultaneously disrupting malaria transmission at different points in the parasite life cycle makes sense. Interventions that use effective drugs to decrease parasite populations and interventions that use effective insecticides to decrease mosquito-human interactions could complement each other, reducing the number of infectious mosquito bites in a community faster and to a higher degree than either strategy used alone [25, 26]. Nonetheless, decisions about when, where, and how to use each strategy (including whether to use them simultaneously in the same communities) will depend on national programmes’ goals and should be tailored to local context.

This will require, among other things, an expanded evidence base built upon impact evaluations from various disease ecologies and transmission intensities. Developing this evidence base is a challenge, however, as the most robust methods for generating evidence (e.g., cluster randomized trials) are often unrealistic—the time, resources, and expertise required, and limited space available, are prohibitive. Observational/ecological studies can help address these challenges by allowing the geographical and temporal linking of malaria intervention coverage data, ecological and environmental data, and data from routine surveillance activities in order to provide insight into how trends in malaria incidence change in response to various interventions or packages of interventions [11, 27]. Observational research questions are also adaptable to the unique malaria control landscape that exists in each country, taking advantage of ‘natural experiments’ that result from programme implementations.

Here, an observational analysis of one such natural experiment from the Ségou Region of Mali in 2014 is described. That year, Bla and Barouéli Districts both received IRS with a micro-encapsulated formulation of the organophosphate insecticide pirimiphos-methyl (Actellic®300CS) as part of the US President’s Malaria Initiative (PMI) Africa Indoor Residual Spraying (AIRS) project. Also, in 2014, Ségou was in the midst of expanding a pilot programme to provide SMC with SP + AQ to children aged 3 to 59 months in Bla and San Districts. The timing of these interventions presented a unique opportunity to analyse the impact of both tools, deployed individually and in combination, across neighbouring districts using quality-assured passive surveillance data.

## Methods

### Study setting

**Fig. 1:**
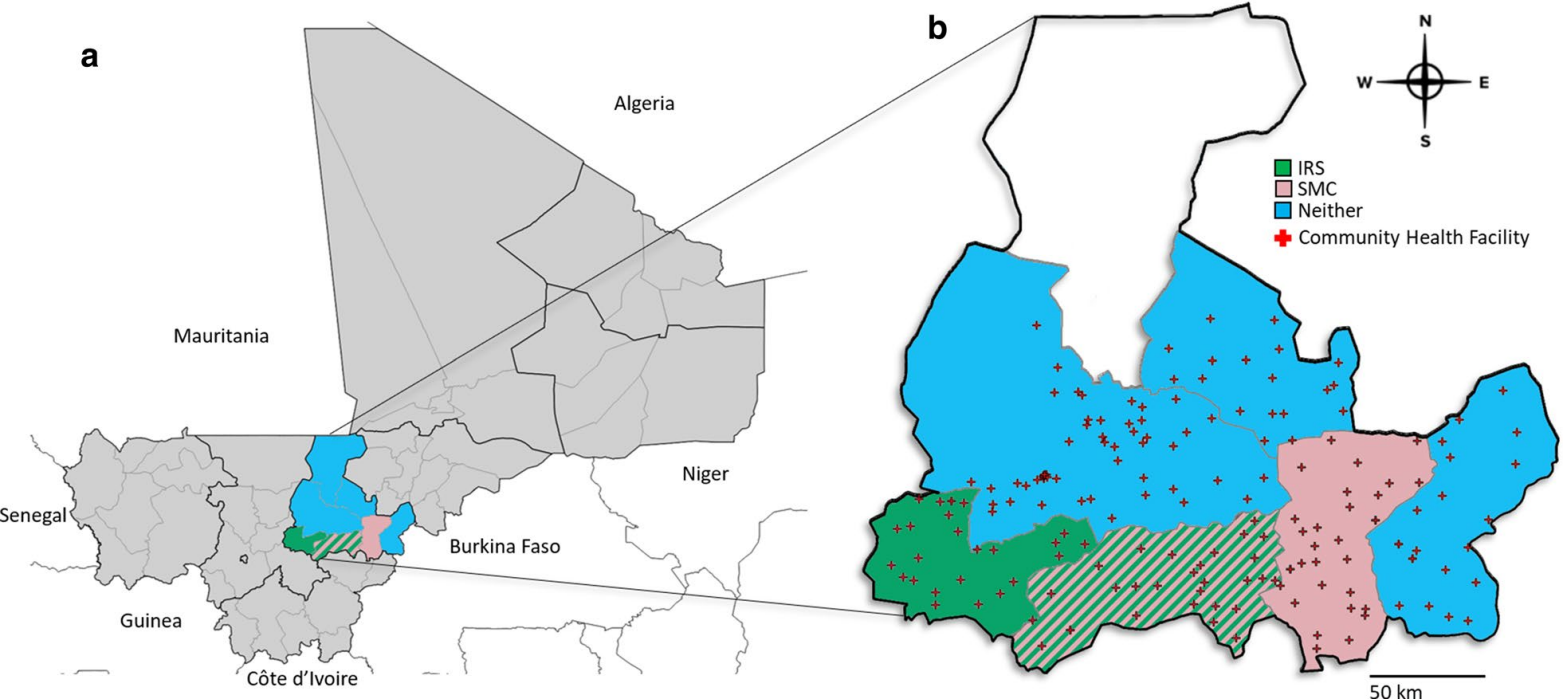
Study site. **a** The location of Mali in West Africa, with Ségou Region highlighted. **b** The locations of the community health facilities in Ségou that reported malaria rapid diagnostic test results during the months analysed here, with the indoor residual spray (IRS) and seasonal chemoprevention (SMC) status of each district indicated

Figure 1 illustrates the location of study site in Mali, which has been previously described [11]. The primary malaria vector in Ségou is *Anopheles gambiae *sensu lato (*s.l*.), and resistance to pyrethroids is well documented in this population [28, 29]. The six districts analysed here have also been shown to be similar to one another with respect to population density, rainfall patterns, malaria transmission seasonality, and population-adjusted *Plasmodium* prevalence rates [30]. Also similar across districts were the proportion of suspected malaria cases presenting to the health system that received a rapid diagnostic test (RDT; 90%), the proportion of RDT-positive patients that received an appropriate artemisinin-based combination therapy (ACT; 69%), and overall health facility reporting rates (> 98%) [11]. Though district resolution indicators are not available, all districts in Ségou Region benefited from a policy targeting universal coverage with intermittent preventive treatment in pregnant women and regional reports indicate high levels of LLIN ownership (92% of surveyed households reported owning at least one LLIN in 2013) and use (73% of survey respondents reported having slept under an LLIN the previous night in 2013) [31]. In the context of this study, without district-level indicators available, these regional estimates are assumed to be similar across all districts in the study area.

### IRS intervention

IRS with Actellic® 300CS was implemented in Barouéli and Bla Districts with support from the PMI AIRS project [32] (Table 1). The 2014 campaign began on July 15 and ended on August 25, reaching 96.5% coverage of targeted houses in Barouéli (279,441 people protected, greater than 93% of the population) and 98.2% coverage in Bla (334,115 people protected or 95% of the population) [32].

**Table 1:** Summary of indoor residual spraying (IRS) and seasonal malaria chemoprevention (SMC) in Ségou Region, 2014

Intervention	District	IRS implementer	IRS coverage (eligible structures sprayed)	IRS coverage (total population protected)	SMC implementer	SMC 1 coverage (children)	SMC 2 coverage (children)	SMC 3 coverage (children)
IRS	Barouéli	PMI	61,234 (97%)	279,441 (93%)	–	–	–	–
SMC	San	–	–	–	UNICEF	79,124 (99%)	74,391 (93%)	81,343 (102%)
IRS + SMC	Bla	PMI	96,229 (98%)	334,115 (95%)	NMCP	69,132 (102%)	70,988 (105%)	-
Neither	Macina	–	–	–	–	–	–	–
Neither	Niono	–	–	–	–	–	–	–
Neither	Ségou	–	–	–	–	–	–	–

### SMC intervention

SMC was implemented in Bla and San Districts with support from the ACCESS SMC project and UNICEF [33] (Table 1). The target population was all children aged 3 to 59 months, with a monthly course of SP + AQ for 4 months of the rainy season, beginning each August. In Ségou Region, SMC was first piloted in San District in 2013, and 94.2% of the target population received two rounds. In 2014 the programme expanded to include both Bla, where 105% of the target population received two rounds, and San, where 102% of the target population received three rounds. The number of children who received SMC exceeded the estimated population in several localities, leading to a coverage above 100%.

### Estimation of malaria incidence rates

Datasets were organized, cleaned, transformed, and joined using Microsoft Excel 2016 with Power Query v2.41 (Microsoft Corp, Redmond, WA, USA) and Tableau v10.0 (Tableau Software Inc, Seattle, WA, USA). Descriptive statistics were calculated using Excel 2016 and Tableau v10.0. Malaria incidence rates were estimated as previously described [11], using RDT-confirmed cases of *Plasmodium falciparum* malaria reported in the Système Numérique d'Information Sanitaire Intégré (SNISI) with health facility catchment area and district population estimates obtained from the Ministère de la Santé de la République du Mali (Ministry of Health), Direction Régionale de la Santé.

During the months analysed here (January 2014 to March 2015), there were 2611 reports from 171 different health facilities across the six districts (for the purposes of this analysis, data from the health district of Markala is reported and analysed as part of Ségou District, the administrative unit to which it corresponded in 2014). 421,964 total RDT + test results were reported, with 78% of all cases being reported during June to January, corresponding to seasonal rainfall patterns [11]. District reporting rates were greater than 98% for all districts, although facility-months in which no data were reported were censored from analysis (less than 2% of the total; range of 0.6% to 3.8% across intervention strata). Of additional note, MEASURE Evaluation had been actively assisting the Ministry of Health since 2012 with SNISI data quality assurance activities at all levels of the system in Ségou Region, including tracking commodity stockouts.

### Observational analysis of the impact of malaria interventions on incidence rates

To analyse monthly trends in malaria incidence, a quasi-experimental time series approach was used. Districts were stratified by intervention status (Table 1): SMC (San District; total population 394,000), IRS (Barouéli District; total population 236,000), IRS + SMC (Bla District; total population 334,000), and control districts receiving neither IRS nor SMC (Macina, Niono, and Ségou Districts; total population of 1.42 million). The total number of RDT + test results reported were aggregated accordingly and epidemiological curves showing the incidence of RDT-confirmed malaria cases per 10,000 person-months were plotted by calendar month for each IRS stratum. Separate analyses were performed for the total all-ages population and for the under 5 year old population (u5).

To describe the seasonal impact of each intervention, the cumulative incidence of RDT + malaria cases observed from September to February was calculated in each intervention district and compared to the cumulative incidence reported from the same time period in the control districts using a crude percent reduction in number of incident cases per 10,000 population. This time period corresponds to both the high transmission season and the months immediately after the completion of IRS and/or the first round of SMC administration. To help further describe estimates of impact, a simple negative binomial regression model was used to calculate incidence rate ratios (IRR) comparing the cumulative number of incident cases per 10,000 person-months at risk from each health facility in each intervention district to each health facility in the non-intervention control districts, using robust standard errors clustered at the district level.

A monthly protective efficacy estimate for each intervention package was calculated using the percent incidence reduction observed in intervention districts relative to control districts:

$$\left( {{\text{Incidence}}_{{{\text{control}}}} - {\text{ Incidence}}_{{{\text{intervention}}}} } \right)/{\text{Incidence}}_{{{\text{control}}}} {\text{x 1}}00\%$$

To describe potential interactions between SMC and IRS, and how this interaction might change over time, the protective efficacy estimates observed in the combined intervention district (IRS + SMC) were compared to the protective efficacy estimates expected assuming each intervention had a completely independent effect, following the methods of VanderWeele and Knol [34]. As such, a ratio of observed effect to expected effect was calculated:

1. Expected = Efficacy_IRS_ x (1-Efficacy_SMC_) + Efficacy_SMC_
2. Ratio = Observed (Efficacy_IRS+SMC_): Expected (Efficacy_IRS_ + Efficacy_SMC_)

A synergistic additive effect is suggested if the above ratio is greater than 1, an independent additive interaction if the ratio = 1, and an antagonistic additive effect is suggested if the ratio is less than 1.

## Results

Table 2 shows a summary of all the RDT + confirmed malaria cases reported from the six districts analysed here, from September 2014 to February 2015. There were 260,661 total RDT + test results during this time period, with 131,260 (50%) coming from the u5 population. RDT + case incidence was four times higher in the u5 population (2753 per 10,000) compared to the over 5 year old population (679 per 10,000).

**Table 2:** Summary of positive rapid diagnostic test (RDT +) results from study districts, Sep 2014–Feb 2015

	Total RDT + fevers	Total population	Estimated cumulative cases per 10,000 persons; Sep 2014 through Feb 2015
All Ages	260,661	2,383,916	1093
Under 5	131,260	476,783	2753
Over 5	129,401	1,907,133	679

**Fig. 2:**
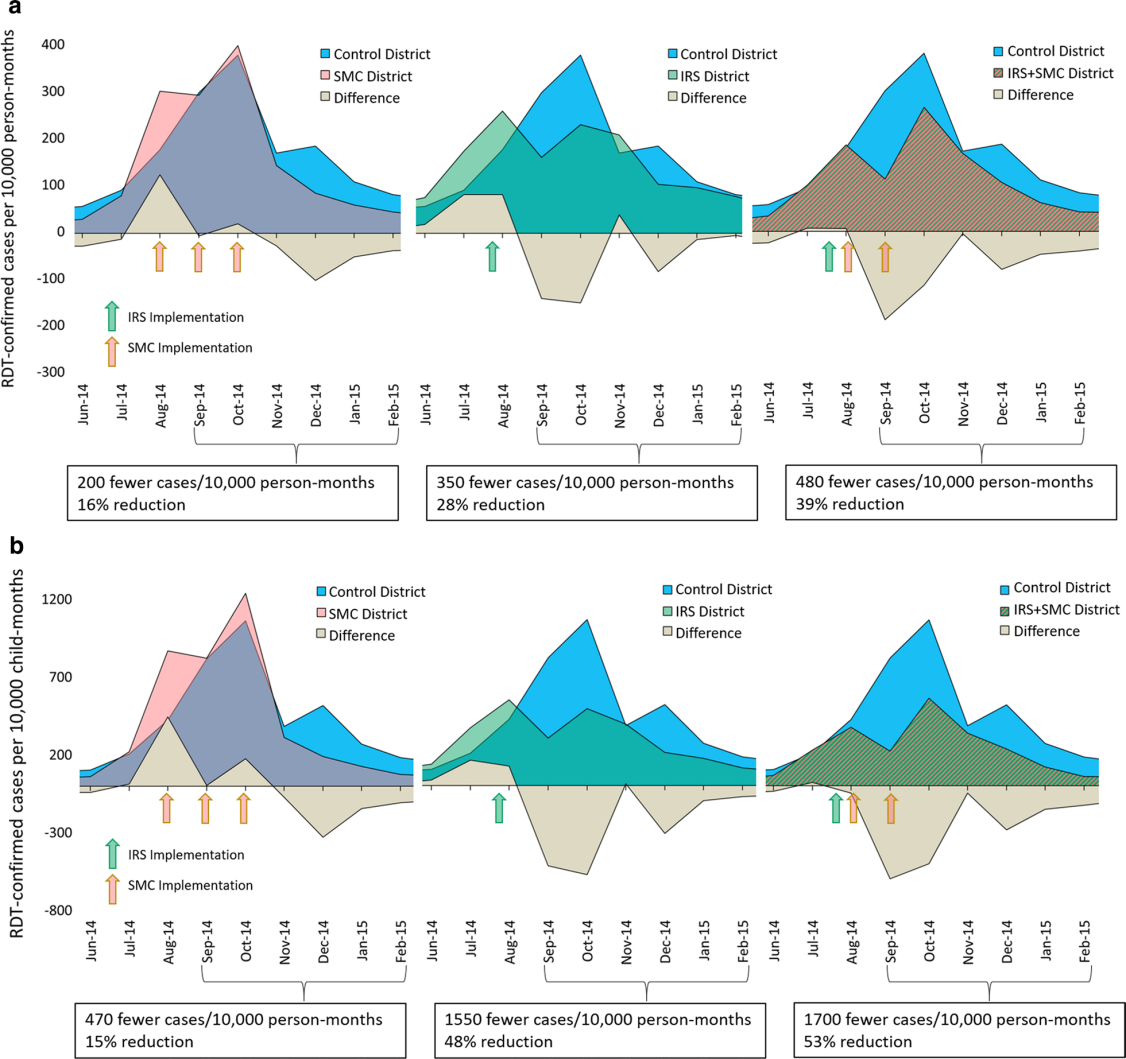
Monthly incidence of confirmed malaria cases in intervention districts relative to the control districts. Epidemiologic curves for each intervention district are overlaid on the contemporary curves from the three neighboring districts that received neither intervention in 2014 (control districts; in blue). The area of the tan curves shows the difference between the incidence rates observed in each intervention district relative to the control districts, illustrating the overall impact of the interventions on rapid diagnostic test confirmed (RDT +) malaria case rates. Results are presented for (**a**) the total all-ages population and (**b**) the population under age 5

Cumulative incidence rates associated with each intervention package are presented in Table 3. For each age group, the largest incidence reduction was in the IRS + SMC district. Figure 2 illustrates the corresponding epidemiologic curves for June 2014 to February 2015. Shown is the monthly incidence of RDT + test results per 10,000 person-months at risk from control districts compared to the intervention districts. In each case, during the 6 months of peak malaria transmission, incidence was lower in the intervention districts than in non-intervention control districts (Fig. 2).

**Table 3:** Cumulative confirmed case incidence rates (Sep 2014–Feb 2015) stratified by intervention package

	Control incidence (No SMC or IRS)	SMC incidence (% reduction)	IRS incidence (% reduction)	IRS + SMC incidence (% reduction)
All ages	1226	1030 (16%)	883 (28%)	752 (39%)
Under 5	3218	2758 (15%)	1682 (48%)	1529 (53%)
Over 5	653	538 (18%)	631 (3%)	498 (24%)

Incidence rates are shown in cases per 10,000 person-months at risk

*SMC* Seasonal Malaria Chemoprevention; *IRS* Indoor Residual Spray

The average 6 month cumulative incidence rate in the all-ages population was 1226 cases per 10,000 person-months at risk in the control districts. The all-ages incidence was lower in each of the intervention districts: 16% lower in the SMC district (1030 cases per 10,000); 28% lower in the IRS district (883 cases per 10,000); and 39% lower in the IRS + SMC district (752 cases per 10,000). Trends were similar in the u5 population, in which the incidence was 3218 cases per 10,000 child-months at risk in the control districts and was 15% lower in the SMC district (2758 cases per 10,000); 48% lower in the IRS district (1682 cases per 10,000); and 53% lower in the IRS + SMC district (1529 cases per 10,000).

The cumulative IRRs calculated for each intervention district indicate the same trends. In the target u5 population, the IRR for SMC only was 0.84 (95% CI 0.45–1.55; *p* = 0.576), for IRS only it was 0.48 (0.28–0.80; *p* = 0.005), and for the combination of IRS + SMC it was 0.47 (0.24–0.90; *p* = 0.024). In the all-ages population, the estimated IRRs were 0.80 (0.59–1.09; *p* = 0.154) for SMC only, 0.72 (0.54–0.96; *p* = 0.023) for IRS only, and 0.64 (0.46 – 0.91; *p* = 0.011) for the combination of IRS + SMC.

**Fig. 3:**
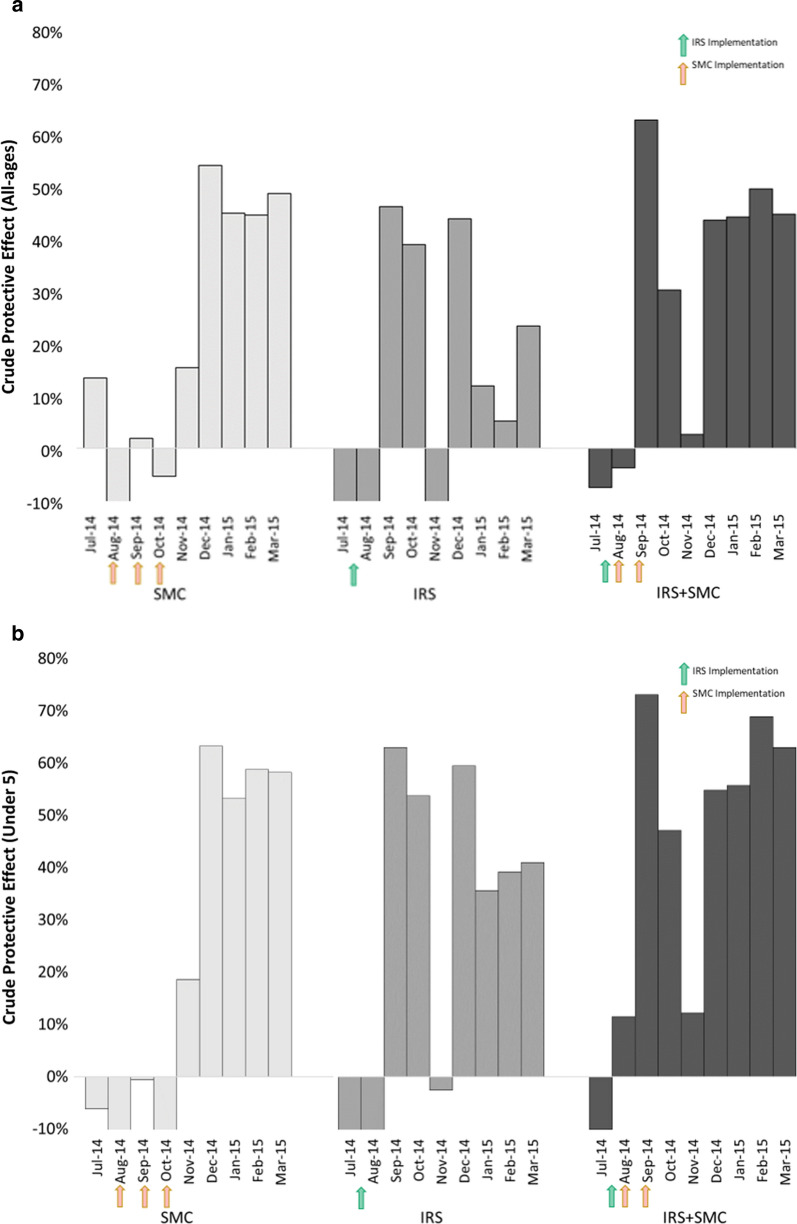
The monthly protective effect of each intervention package. The protective effect is the reduced incidence observed in each district as a percentage of the total incidence observed in the non-intervention comparator districts. Results are shown for (**a**) the total all-ages population and (**b**) the population under age 5

Looking at the estimated protective effect of each intervention package by month, shown in Fig. 3, suggests that the SMC-only intervention had a more moderate effect initially (15% fewer all-ages cases and 19% fewer u5 cases in November, the first month after the last SMC dose was administered) that increased over time, approaching 50% (all-ages) to 60% (u5) protective efficacy by December and remaining there at least until February. The IRS-only intervention had a rapid, comparatively large impact (46% fewer all-ages cases and 63% fewer u5 cases in September, the first month after completion of the IRS campaign) that more noticeably diminished over time – an observation in line with standard cone bioassay test results from 2014 that indicated a residual efficacy (test mosquito mortality greater than or equal to 80% at 24 h post-exposure) of three months in Ségou [28]. The impact of the combined interventions was both rapid (63% fewer all-ages cases and 73% fewer u5 cases in the first month) and of longer duration, remaining relatively high (near 50% for the all-ages population and near 60% in the u5 population) for at least 6 months, until February.

It is worthwhile to try to describe the nature of the additive effect between IRS and SMC that was observed. The nature of the interaction seems to have changed as the transmission season progressed, as shown in Table 4. In both age groups, the ratio of observed impact to expected impact (assuming an independent additive interaction, see methods) is greater than 1 initially, indicating that during the first months the effect may be synergistic. Interestingly, this synergism declines by December and rebounds by February in each age group. The average effect over the 6 months is close to 1, indicating an overall effect similar to that of an independent additive interaction.

**Table 4:** Interaction ratios for observed effects compared to expected effects

	All ages			Under 5		
	IRS + SMC	IRS + SMC	Interaction ratio	IRS + SMC	IRS + SMC	Interaction ratio
Month	Expected impact (%)^1^	Observed impact (%)	Observed/expected	Expected impact (%)^1^	Observed impact (%)	Observed/expected
14-Sep	47	63	1.33	63	73	1.17
14-Oct	36%	30	0.85	46	47	1.02
14-Nov	4%	3	1.55	16	12	0.74
14-Dec	74%	44	0.59	85	55	0.64
15-Jan	52%	44	0.86	70	56	0.8
15-Feb	47%	50	1.05	75	69	0.92
Average	43%	39	0.9	59	52	0.88

^1^Efficacy_IRS_ x (1-Efficacy_SMC_) + Efficacy_SMC_

*SMC* Seasonal Malaria Chemoprevention, *IRS* Indoor Residual Spray

## Discussion

Though observational studies have limitations, the results presented here suggest a possible combined effect for the co-implementation of IRS and SMC in central Mali. Compared to neighboring districts that received neither intervention, routinely reported confirmed malaria case incidence rates were reduced by the greatest proportion in Bla (53% reduction in u5 cases, 39% reduction in all-ages cases), where IRS and SMC were both implemented in 2014, compared to in Barouéli, where IRS alone was implemented (48% reduction in u5 cases, 28% reduction in all-ages cases) and San, where only SMC was implemented (15% reduction in u5 cases, 16% reduction in al-ages cases). The IRR estimates show similar trends, indicating that the impact of SMC was less evident (0.84, *p* = 0.576 in the u5 population, 0.80, *p* = 0.154 among all-ages) than the impact of IRS alone (0.48, *p *= 0.005 among u5s, 0.72, *p* = 0.023 among all-ages) or the impact of IRS + SMC (0.47, *p *= 0.024 among u5s, 0.64, *p* = 0.011 among all-ages). In both age groups, the impact of IRS and IRS + SMC were both highly statistically significant, though overlapping IRR confidence intervals indicate that the overall cumulative trends towards greater reduction in the combined intervention district compared to the IRS only intervention district were not statistically significant.

One limitation of this study worth noting is that it was not designed specifically to estimate the impact of SMC, and health facility case incidence rates may not be the best outcome to measure the effect of this intervention. Nonetheless, an impact for SMC at the community level was evident even without 100% coverage of the target population with the recommended four rounds of treatment—which was not yet achieved in the 2014 pilot. It is likely that subsequent SMC campaigns in Ségou Region achieved better impact as the programme matured, coverage rates improved, and more children from more districts were included.

Other key limitations of this analysis include lack of district-specific supportive data that would help control for potential confounding and/or co-variable factors, and the possibility that intervention and non-intervention districts may be fundamentally different in ways not understood here. Other weaknesses include the limited geographical and time ranges analysed: this work is taking advantage of a very time-limited confluence of events and describes only what happened in one region of central Mali in 2014. It would be important to examine evidence of interactions between IRS and SMC in other countries and from other years, including comparable pre-intervention malaria trends, if and where datasets become available. Also interesting would be identifying opportunities to examine interactions between IRS and other drug-based interventions, particularly mass drug administration campaigns [35–38], as well as opportunities to more fully explore the differential impact of intervention packages in the over-5 population, as presented in Table 3. Though the impact of SMC observed here in the over-5 population (18% reduction in reported case incidence) was greater than expected, and indeed similar to the impact observed in the target u5 population (15% reduction in reported case incidence), the impact of IRS was not evident in the over-5 population (a 3% reduction in reported case incidence vs. a 48% reduction in the u5 population). Why the effect of IRS observed here was much more substantial in the u5 population than in the over 5 population is unclear, though it is likely that the underlying malaria burdens and health care utilization rates, as well as other important factors, also differed substantially across these two populations and are influencing these estimates. Further work investigating any differential impact of vector control across different segments of the population could include active measurements of infection incidence rates as well as key anthropological and behavioral data to help clarify.

Nonetheless, the overall results presented here are in line with a recent study in Senegal which similarly found reductions in incidence greatest in areas that received both IRS and SMC (52% reduction) compared to areas that received IRS (38% reduction) or SMC (32% reduction) alone [27] and recent modeling studies that indicate a high probability of strong synergies between complementary IRS and population-based drug interventions such as mass drug administration [25, 26].

Results also suggest that in 2014 in Mali, IRS with Actellic was fast-acting—preventing many cases of malaria in the months immediately after the spray campaign—but peak efficacy had a relatively short duration. In contrast, SMC did not make an immediate impact on malaria cases presenting to the health system, but the effect strengthened over time and lasted for at least 6 months. While the exact nature of the combined effect for using both interventions at the same time remains difficult to define, it is interesting to point out that the IRS + SMC intervention seemed to combine the best of both effects: acting quickly to reduce malaria cases initially and maintaining a relatively high protective effect for the duration of the study. Furthermore, the nature of the interaction seemed to change over time, initially showing evidence of a true synergistic effect that lessened over time. Both of these observations might make sense considering their different mechanics. The impact of IRS is expected to wane over time as the residual efficacy of insecticide on interior wall surfaces naturally decreases, while the effect of an SMC campaign might be expected to increase with each subsequent round as the number of treatments in the population increases. These are important factors that might impact the planning and timing of combined malaria control interventions, both relative to one another and to expected transmission seasons, and merit further study.

Further evidence for a combined impact of IRS and SMC used together comes from the experience in Bla District in 2015, when removing IRS led to a significant increase in malaria transmission that year despite four rounds of SMC [11]. This is likely to inform our understanding of when it might make sense to stop spraying in an area, especially as population-based drug interventions become more widely used.

Despite the prospect of a complementary effect for combining 3GIRS and mass drug administration campaigns, it is important to ask when such combined strategies would make sense programmatically, especially in terms of resource allocation and marginal gains. It is likely that the answer will differ according to underlying transmission intensity [5]. For example, in areas of low and very low transmission, using multiple interventions at the same time in key hotspot communities could be a cost-effective way to accelerate progress toward elimination (provided, of course, that enhanced surveillance and treatment capacity is maintained). In areas where the disease burden is still relatively high and/or widespread across many districts, there may be a case for achieving a larger impact by using available additional interventions across different communities, expanding the footprint of programmatic activities and likely preventing more cases of malaria overall and achieving greater cost-effectiveness by reaching more people.

## Conclusions

One of the key elements in the Global Strategic Framework for Integrated Vector Management [39] is ensuring that there is “adequate, evidence-based guidance on combining IRS with LLINs and other malaria control interventions.” The analysis presented here, while limited in scope, suggests that combining IRS and SMC in a high-burden area of central Mali with high rates of LLIN access and pyrethroid-resistant vectors had a greater impact on reducing malaria case incidence than the use of either intervention individually.

## Data Availability

Intervention, entomology, and climate datasets used and/or analysed during this study were consolidated from public sources and are available from the corresponding author upon reasonable request. Requests about the malaria surveillance datasets analysed here should be directed to IC (idrissaciss68@yahoo.fr): Director, Programme National de Lutte contre le Paludisme, Bamako, Mali.

## Abbreviations

3GIRS: Third generation indoor residual spraying
ACT: Artemisinin-based combination therapy
AIRS: Africa Indoor Residual Spraying
IRS: Indoor residual spraying
LLIN: Long-lasting insecticidal nets
PMI: US President’s Malaria Initiative
RDT +: Positive rapid diagnostic tests
SMC: Seasonal malaria chemoprevention
SNISI: Système Numérique d'Information Sanitaire Intégré
SP + AQ: Sulfadoxine-pyrimethamine plus amodiaquine
u5: Children under 5 years old
WHO: World Health Organization
